# *Self-Regulation Questionnaire (SRQ)* in Spanish Adolescents: Factor Structure and Rasch Analysis

**DOI:** 10.3389/fpsyg.2018.01370

**Published:** 2018-08-10

**Authors:** María Carmen Pichardo, Francisco Cano, Angélica Garzón-Umerenkova, Jesús de la Fuente, F. Javier Peralta-Sánchez, Jorge Amate-Romera

**Affiliations:** ^1^Department of Educational and Evolutionary Psychology, University of Granada, Granada, Spain; ^2^School of Psychology, Fundación Universitaria Konrad Lorenz, Bogotá, Colombia; ^3^Department of Psychology, School of Psychology, University of Almería, Almería, Spain; ^4^Universidad Autónoma de Chile, Santiago, Chile; ^5^Doctorate in Psychology, University of Almería, Almería, Spain

**Keywords:** self-regulation questionnaire, Rasch model, validity, self-regulation measurement, adolescent

## Abstract

**Background:** The Self-Regulation Questionnaire (SRQ) is an instrument employed to measure the generalized ability to regulate behavior. Self-regulation is related to the management of risk behaviors, such as drug abuse or anti-social behaviors. The SRQ has been used in young adult samples. However, some risk behaviors are increasing among adolescents. The aim of this study is to examine the psychometric properties of the SRQ among Spanish adolescents.

**Methods:** 845 high-school Spanish students (*N* = 443; 52.43% women), from 12 to 17 years old and ranging from the first to the fourth year of studies, completed the SRQ. A confirmatory factor analysis (CFA) was carried out in order to establish structural adequacy. Then, a study of each subscale was conducted using the Rasch model for dimensionality, adjustment of the sample questions, functionality of the response categories, and reliability.

**Results:** While controlling for method effects, the data showed goodness of fit with the four-factor solution and 17 items (Goal setting, Decision making, Learning from mistakes, and Perseverance), and the four sub-scales were unidimensional according to the Rasch analysis. The Rasch model itself was shown to be reliable, but not at the level of persons. This means that the instrument was not sensitive enough to discriminate people with different self-regulation levels.

**Discussion:** These results support the use of the Spanish Short SRQ in adolescent samples. Some suggestions are made to improve the instrument, particularly in its application as a diagnostic tool.

## Introduction

Various authors have identified self-regulation as the capacity to manage and demonstrate appropriate behaviors, considering it a cyclical process that consists of three components: forethought, performance control, and self-reflection (Zimmerman, 2000; Panadero, 2017). Similarly, it is considered one of the most important psychological variables for adequate personal, social, and academic development during adolescence (Helle et al., 2013; Kalimulin et al., 2016).

The regulation of one’s own conduct is key to adequately developing and maintaining healthy habits, and avoiding becoming involved in risk behaviors – such as the consumption of alcohol or other drugs. There are other factors that can act as motivators of these healthy habits, but it is unlikely that these factors will produce long-lasting behavioral changes unless the subjects develop the means to exercise control over their motivation and their behavior related to health (Bandura, 2005). Hence, the importance of developing, during adolescence, adequate self-regulation, which will act as a resilience factor in confronting the situations of risk that are so common at this age.

### Self-Regulation as a Health-Promoting Variable During Adolescence

Various different studies have evidenced the relationship between self-regulation and different behavorial problems, both internalized and externalized. In this vein, a lack of self-regulation has been related to anxiety, depression, aggressive conduct, bullying, and delinquency (Muris et al., 1999; Beauchaine et al., 2007; de la Fuente et al., 2009; Brooks et al., 2010; Garner and Hinton, 2010; Tavakolizadeh and Ebrahimi-Qavam, 2011; Rhodes et al., 2013; White et al., 2013).

Nevertheless, the large majority of studies on the importance of self-regulation have focused on addictive disorders linked to gambling and substance consumption (Madden et al., 1997; Hull and Slone, 2004), and alcohol consumption in particular (Brown et al., 1999; Carey et al., 2004; Pearson et al., 2013). These effects are especially relevant in adolescence and youth, two stages of development characterized by the search for personal identity, the distancing of oneself from the family environment and connections with one’s peer group. In this regard, self-regulation might act as an index of the resilience of adolescents in situations of greater psychosocial risk (Dishion and Connell, 2006; Artuch-Garde et al., 2017).

The effects of alcohol and drug consumption during early and middle adolescence are truly worrying, and are linked with health problems (Chaves et al., 2013), problems at school (Ekberg et al., 2016), mental disorders (Borges et al., 2017), unprotected sex (Remy et al., 2013; Boyer et al., 2017), and delinquency (Doherty et al., 2008). As in many countries, in Spain, alcohol consumption is very high among adolescents. A study undertaken by OEDA (the Spanish Drugs and Addiction Monitoring Centre of the Ministry of Health Social Services Equality, 2016), shows that during 2014, 78.9% of students in secondary education between the ages of 14 and 18 had habitually consumed alcohol during the previous month, placing the first intake at 13.8 years old. Similarly, the percentage of schoolchildren that had had acute alcohol poisoning was 33.1%, and the percentage of those who had drunk to excess (binge-drinking) was 47.3%.

Wills and Stoolmiller (2002) found that good self-regulation skills (soothability, dependability, planning, and problem solving) were negatively associated with substance use among sixth graders; whereas poor regulation (impatience, distractibility, and being easy to anger) was positively associated with substance use among sixth graders and predicted increases in levels of substance use over the subsequent 3 years. Likewise, Bower et al. (2012) – in a study carried out on the relationships between risk and protective factors and school experiences for three adolescent groups aged 12–18 years old (including: 31 early-onset offenders who began offending before the age of 12; 36 late-onset offenders who began offending at or after 12 years of age; and 36 who were non-offenders) – found that self-regulation, understood as goal-setting, planning, and self-reflection, builds resilience within the domains of school, peers/leisure, and self. Along these lines, Dishion and Connell (2006) consider that although negative experiences of school, individual traits, and associating with antisocial peers can influence adolescents to develop antisocial behavior, these negative influences can be mediated by self-regulation. Adolescents who do not have adequate self-regulation do not tend to plan their behavior, they do not have any set goals, and neither do they control the degree to which their conduct brings them closer to these goals. Rather, they act impulsively: which can have very worrying results, both academically and in the personal or social sphere (e.g., Eisenberg et al., 1996; King et al., 2013).

Most of these studies champion the critical role that the regulation of negative emotions in situations of frustration has for appropriate personal and social development. This ability to self-regulate allows adolescents to adequately avoid and confront problems related to the consumption of toxic substances, alcohol, or involvement in antisocial behavior: hence, the importance of having reliable and valid measures that enable the evaluation of self-regulation during adolescence.

### Evaluation of Self-Regulation: Self-Regulation Questionnaire (SRQ)

The SRQ, developed by Brown et al. (1999), evaluates subjects’ self-regulation of behavior, understood as the ability to plan and manage their own behavior in a flexible way, according to the desired outcomes. Although the questionnaire has been adapted to educational contexts, it was initially designed within the field of addictive behaviors. The authors, using squared multiple correlation coefficients, carried out an initial design for 63 items (26 reverse) that constituted 7 scales: (1) *informational input*, which refers to the ability of a person to obtain information from their environment on their current state; (2) *self-evaluation*, for which the information is used in comparison with personal goals, rules and expectations; (3) *instigation to change*, wherein the person perceives whether or not there are discrepancies between their current state and their desired state; (4) *search for alternatives*, with the aim of reducing discrepancies; (5) *planning for change*, referring to the strategies or actions for carrying out the process of change; (6) *implementation of strategies for change*; and (7) *goal attainment evaluation plan*. The instrument, in its English version, has mainly been used with university students.

Different studies have analyzed the SRQ’s psychometric properties, establishing several factorial solutions. Carey et al. (2004), using a sample of 391 American undergraduate students, ranging in age from 17 to 24, establish a unifactorial solution composed of 31 items, which led the authors to propose a new measure: the Short SRQ (SSRQ), with a correlation of *r* = 0.96 between the two versions (suggesting that the short version appears to be a good alternative to the full-scale one). Subsequently, Neal and Carey (2005), again using undergraduate students, verified the factor structure and internal consistency of the 31-item SSRQ. Using a confirmatory factor analysis (CFA), they did not find goodness of fit between the data using all of the SSRQ items. Nonetheless, they obtained a bifactorial solution, with 11 items loaded significantly on the first factor (Impulse Control), and 10 loaded significantly on the second factor (Goal Setting). Potgieter and Botha (2009), with a sample of undergraduate students (*N* = 385) at the University of South Africa, analyzed the factorial structure of the SSRQ and proposed a solution of seven factors and 28 items, using a principal component analysis that explained 61.79% of the total variance: Monitoring; Decision-making; Learning from mistakes; Perseverance; Self-evaluation; Creativity; and Mindful awareness.

In Spain, Pichardo et al. (2014) used the SRQ (Brown et al., 1999) and studied the fit for each of the proposed factorial models (Brown et al., 1999; Carey et al., 2004; Neal and Carey, 2005; Potgieter and Botha, 2009). None of the models showed goodness of fit, and the authors proposed a short version of the SRQ for the Spanish context, the *Spanish Short Self-Regulation Questionnaire* (SSSRQ), with a structure of 17 items grouped in four factors (Goal-setting, Perseverance, Decision-making, and Learning from mistakes). However, in this study, the authors found that although the indices and statistics showed a good fit, they proceeded to establish a relation between the errors of two items because they were written in a negative sense. Later, the modified model was analyzed using the exploratory sample (ESm); both the fit indices and statistics show that the modified model fits better than the initial one. In this line, several authors such as Tomás et al. (2010), consider that one potential bias of the method that has been proposed, and has been evaluated in the literature, consists of the appearance of method factors method that are associated with negatively formulated items. It has been widely used in psychology in order to avoid acquiescence bias for both positively and negatively formulated items. Nonetheless this formulation, as has been highlighted by these authors, could complicate the factorial analysis of the scales.

More recently, Garzón Umerenkova et al. (2017) have studied the pychometric properties of the SSSRQ with Rasch analysis. The results showed goodness of fit with the proposed factorial structure, and some changes were recommended to improve the measurement of the degree of ability for each factor.

### Rasch Analysis

Rasch analysis tests data against a measuring model in order to determine the degree to which the data fit the model’s expectations for building the measure (Smith, 2012). This type of analysis is basically built upon two principles: unidimensionality and local independence. Unidimensionality enables the estimation of the existence of a unique principal factor of the instrument, and local independence shows that people’s responses to any question are independent of their response to another question. Using the logit scale, the model represents the ability of the individual, who responds to test items at different magnitudes of difficulty (Bond and Fox, 2012).

This study uses Rasch analysis to examine the psychometric properties of the SSSRQ. Therefore, *ability* should be interpreted as the attribute “self-regulatory capacity,” according to the specific component that measures each of the subscales and understanding that each subscale refers to a different attribute (Goal setting, Perseverance, Decision making, and Learning from mistakes).

### Objectives

The SSSRQ has been used mainly in the study of self-regulation and its relation to addictive behaviors, focusing on the adult population, particularly university students. However, addictive behaviors (alcohol, drugs, mobile phone use, social networks, etc.) are especially important during adolescence. Therefore, it would be extremely useful to provide instruments for the evaluation of self-regulation, with adequate consistency and validity for the target population.

On the one hand, the aim of the research is to analyse the factorial structure of the SRQ for the Spanish population in a sample of secondary school students through CFA. On the other hand, the research seeks to provide an analysis of the psychometric properties of the questionnaire using Rasch analysis to check: the dimensionality; the fit of the items to the model; the functioning of the measurement scale; the construct validity; the reliability; and the differential item functioning (DIF) for each of the test’s four dimensions.

## Materials and Methods

### Participants

A total of 845 students in Secondary Education in the Spanish province of Almería, aged between 12 and 17 years old (*M* = 14; *SD* = 1.29). Out of these, 52.43% (*n* = 443) are female and the rest male (47.57%; *n* = 402). The participants are all within one of the 4 years of compulsory secondary education (**Table 1**).

**Table 1:** Distribution of the participants per academic year.

Year	*N*	%
First	251	30
Second	237	28
Third	238	28
Fourth	119	14
Total	845	100


### Instruments

The study used the SRQ (Brown et al., 1999) translated and adapted by de la Fuente (unpublished). The instrument measures a person’s self-regulation through seven dimensions: information input (e.g., “I usually keep track of my progress toward my goals”); self-evaluation (e.g., “I have personal standards, and try to live up to them”); instigation to change (e.g., “I am willing to consider other ways of doing things”); search (e.g., “If I wanted to change, I am confident that I could do it”); planning (e.g., “Once I have a goal, I can usually plan how to reach it”); implementation (e.g., “I am able to resist temptation”); and plan evaluation (e.g., “I set goals for myself and keep track of my progress”). Each dimension is made up of 9 items, with 63 items in total scored on a 1–5 Likert-type scale (strongly disagree–strongly agree). The items are drawn up in both positive and negative (R), with the latter reversed for the analyses. The items which make up each factor are: information input (1, 8-R, 15-R, 22, 29-R, 36, 43-R, 50-R, and 57); self-evaluation (2-R, 9, 16, 23, 30, 37-R, 44, 51, and 58); instigation to change (3-R, 10-R, 17, 24-R, 31-R, 38, 45-R, 52, and 59); search (4-R, 11, 18, 25, 32, 39, 46, 53, and 60); planning (5-R, 12-R, 19-R, 26-R, 33-R, 40-R, 47, 54, and 61); implementation (6-R, 13-R, 20-R, 27, 34, 41, 48, 55-R, and 62-R); and plan evaluation (7, 14, 21-R, 28, 35, 42, 49, 56, and 63-R).

### Procedure

The test application was carried out in computer classrooms. Students participated in the study voluntarily. Both the students and their parents signed a written consent prior to participation. The protocols were approved by the relevant School Boards and the Committee on Bioethics in Human Research (University of Almería), which managed the project, and all met the requirements of the Code of Ethics in Psychology and the Spanish Data Protection Act.

### Data Analysis

#### Confirmatory Factor Analysis and Reliability

The assumptions for the factorization of the data and descriptives of the items are studied with SPSS (v. 20). The first-order CFA of the SRQ versions was carried out with the Mpluss 7.3 statistical program. The recommended estimation method for the characteristics of this data (Finney and DiStefano, 2006) is weighted least squares mean and variance corrected (WLSMV). The fit of the model was evaluated according to a combination of different criteria (Hu and Bentler, 1999; Marsh et al., 2004): the chi-square statistic, the comparative fit index (CFI), and Tucker–Lewis index (TLI) with values of more than 0.90 being indicative of adequate fit, and values equal to or greater than 0.95 being indicative of ideal fit. The quantitative error measurements used were the root mean square error of approximation (RMSEA, the confidence interval is included at 90%; 90% IC) with values of 0.06 or less. Finally, the chi-squared differences were used as a criterion of comparison of the added models (Bollen, 1989).

In the evaluation of the structural models, three models of each of the SRQ versions were tested (Tomás et al., 2010) and the correlated trait-correclated method (CTCM) used to model the method effect (negatively formulated items), as recommended by Tomás et al. (2000).

Lastly, the characteristics of the data meant that it was advisable to study the internal consistency through the composite reliability index (CRI, e.g., Graham, 2006). The variance is explained and the data consistency are obtained following Raykov (2001).

### Rasch Analysis

This analysis was conducted using the Winsteps version 3.72.3 statistical package. First, a goodness-of-fit analysis was carried out on the model, taking into account the dimensionality of each subscale and the fit of each item to the model by subscale. Then, the b parameter was established; the reliability both for persons and for the items; the functioning of the response categories; and, finally, a differential item functioning (DIF) by gender and year.

## Results

### Preliminary Analysis

The testing of the assumptions for the data factorization, through the KMO and Bartlett’s sphericity test, showed that the models of the different SRQ versions proposed (Brown et al., 1999; Carey et al., 2004; Neal and Carey, 2005; Potgieter and Botha, 2009; Pichardo et al., 2014) fulfil the factorization assumptions. However, the multivariate normality study, with Mardia’s coefficient, showed that this normality was not fulfilled in the proposed models (see **Table 2**).

**Table 2:** Assumptions for the factorization of data and multivariate normality.

SRQ	KMO	Bartlett’s sphericity test		Mardia’s coefficient
		
		χ^2^	gl	
63 items, 7 factors	0.822	16,145.52	1953	863.98
31 items, 1 factor	0.841	6908.70	465	160.07
21 items, 2 factors	0.821	4101.45	210	64.56
24 items, 6 factors	0.813	4283.75	253	77.50
17 items, 4 factors	0.756	2500.95	136	46.48


*All the chi-squared tests present *p* < 0.001; KMO, Kaiser–Meyer–Olkin test.*

### Factorial Structure and Internal Consistency

The study of the SRQ factorial structure was conducted according to the recommendations of Tomás et al. (2010). It was initiated by examining the factorial structures of the first-order proposals within all the factorial structures derived from the SRQ (both long and short) and continued by modelling an additional method factor for each factorial structure with better fit. Three models were examined in each of the factorial structures proposed by the SRQ and the SSRQ:

• Model 1: model of the baseline of a unique self-regulation factor in each of the proposed factorial structures (long and short versions).
• Model 2: model proposed in each of the propositions and modifications of the SRQ questionnaire: 63 items with seven factors (Brown et al., 1999), 21 items with two factors (Neal and Carey, 2005), 28 items with seven factors (Potgieter and Botha, 2009), and 17 items with four factors (Pichardo et al., 2014).
• Model 3: model that examined an additional method factor in each of the SRQ and SSRQ factorial structures.

The results of the CFA (**Table 3**) show that the data adequately fit the 17-item model with four factors and the additional method-effect factor. On the other hand, the adjustment indices are not adequate for the rest of the factorial structures. In all of the tested versions of the SRQ, model 3 (method effect) showed a significant and greater difference with respect to model 1 than that found between models 2 and 1.

**Table 3:** Goodness of fit for the first-order factorial structures of the Mpluss versions.

Model	χ^2^	*df*	Δχ^2^	Δ *df*	CFI	TLI	RMSEA	90% IC
63 items								
Model 1	10,255.05	1890			0.415	0.395	0.072	0.071–0.074
Model 2 (7 factors)	9955.66	1869	466.31	21	0.434	0.409	0.072	0.070–0.073
Model 3	5490.37	1843	2623.69	47	0.754	0.730	0.048	0.047–0.050
31 items								
Model 1	6320.34	434			0.386	0.342	0.127	0.124–0.129
Model 2	Non-existent	–	–	–	–	–	–	–
Model 3	1675.82	426	1511.28	8	0.870	0.858	0.059	0.056–0.062
21 items								
Model 1	4118.51	189			0.357	0.286	0.157	0.153–0.161
Model 2 (2 factors)	3979.35	188	1805.30	1	0.380	0.307	0.154	0.150–0.159
Model 3	965.92	177	1264.63	12	0.871	0.847	0.073	0.068–0.077
28 items								
Model 1	5740.18	350			0.382	0.333	0.135	0.132–0.138
Model 2 (7 factors)	4272.31	328	997.032	22	0.548	0.479	0.119	0.116–0.122
Model 3	1154.27	318	3363.64	32	0.904	0.886	0.056	0.052–0.059
7 items								
Model 1	2692.53	119			0.357	0.265	0.160	0.155–0.165
Model 2 (4 factors)	2209.44	113	428.71	6	0.476	0.370	0.148	0.143–0.154
Model 3	455.70	104	1410.75	16	0.912	0.886	0.063	0.057–0.069


*All the chi-squared tests present *p* < 0.001; CFI, comparative fit index; TLI, Tucker–Lewis index; RMSEA, root mean square error approximation; CI, confidence interval.*

The proportion of variance explained for the factorial model of the SSSRQ was 86% for all the items. The factors also explained adequate percentages of the variance: goal-setting (90%), learning from mistakes (88%), perseverance (84%), and decision-making (78%). The descriptive analysis and the standardized factor loadings of the items were carried out after reversing the items in negative (5, 6, 12, 13, 19, 21, 33, 40, and 55). These items showed a lower mean (from 2.88 to 3.17) and a larger standard deviation (from 1.14 to 2.21) than the rest. Factorial saturation was significant in all items. Nonetheless, the saturation of some reversed items (items 6, 21, 33, and 40) was larger and only significant with the Method Effect factor, rather than with their own factor (see **Table 4**).

**Table 4:** Descriptives and saturation of the items with their factor from the SSSRQ (original SRQ numeration).

Factor	Item	Statement	*M* (*SD*)	Loading (ME)
F1	1	I usually keep track of my progress toward my goals.	3.48 (1.13)	0.369^∗^
	33	I have a hard time setting goals for myself.	3.08 (2.21)	-0.021 (0.675^∗^)
	40	I have trouble making plans to help me reach my goals.	3.10 (1.23)	0.002 (0.772^∗^)
	42	I set goals for myself and keep track of my progress.	3.23 (1.09)	0.697^∗^
	47	Once I have a goal, I can usually plan how to reach it.	3.49 (1.04)	0.687^∗^
	49	If I make a resolution to change something, I pay a lot of attention to how I’m doing.	3.50 (1.00)	0.677^∗^

F2	6	I get easily distracted from my plans.	2.88 (1.31)	0.070 (0.372^∗^)
	34	I have a lot of willpower.	3.54 (1.16)	0.641^∗^
	41	I am able to resist temptation.	3.18 (1.27)	0.401^∗^

F3	5	I have trouble making up my mind about things.	3.12 (1.27)	0.433^∗^ (0.391^∗^)
	12	I put off making decisions.	3.06 (1.14)	0.386^∗^ (0.316^∗^)
	13	I have so many plans that it’s hard for me to focus on any one of them.	3.13 (1.18)	0.289^∗^ (0.332^∗^)
	19	When it comes to deciding about a change, I feel overwhelmed by the choice.	2.98 (1.17)	0.616^∗^ (0.223^∗^)
	55	Few problems or distractions throw me off course.	2.93 (1.19)	-0.010 (0.345^∗^)

F4	21	I don’t seem to learn from my mistakes.	3.17 (1.34)	0.054 (0.652^∗^)
	28	I usually only have to make a mistake one time in order to learn from it.	3.21 (1.29)	0.649^∗^
	57	I learn from my mistakes.	3.66 (1.16)	0.622^∗^


*F1, goal-setting; F2, perseverance; F3, decision-making; F4, learning from mistakes; ME, saturation of the reversed items in the method effect factor; ^∗^*p* < 0.001.*

The internal consistency of the SSSRQ was 9.97. The CRI also showed adequate internal consistency in the factors of goal-setting (0.95), perseverance (0.87), decision-making (0.84), and learning from mistakes (0.91).

### Goodness of Fit to the Rasch Model

#### Dimensionality

With the understanding that unidimensionality is never perfect, under the Rasch model a series of criteria can be taken into account to establish and discard the possibility of a latent second dimension. Using Rasch Principal Component Analysis of Residuals (PCAR), several criteria can be analysed simultaneously: first, the test measures a dimension when the proportion of variance explained by the measure is ≥40% (Linacre, 2006), moderate when it is ≥30% and an acceptable minimum when it is ≥20%; second, it is necessary to check whether the amount of variance explained by the first contrast is not greater than the amount of variance explained by the difficulty of the items (variance explained by the items); and, third, to discard a second dimension, to see whether the first contrast of residuals is lower than two eigenvalues (Smith, 2012).

**Table 5** shows the results of the analysis of the assumption of unidimensionality for each of the four subscales reported in the AFC of the SSSRQ test: Goal-setting, Perseverance, Decision-making, and Learning from mistakes. Taking the above-mentioned criteria into account, all the subscales present values of the proportion of variance explained by the measure greater than 30%. However, the subscale Goal-setting has a value greater than 2 eigenvalues in the first contrast, which could indicate the presence of a second dimension.

**Table 5:** Variance of standardized residuals for each subscale.

	Eigenvalues	Observed (%)	Expected (%)
Goal setting			
Total raw variance =	8.72	100.00	100.00
Raw variance explained by measures =	2.72	31.2	31.1
Raw variance explained by persons =	0.90	10.4	10.3
Raw variance explained by items =	1.82	20.9	20.8
Raw unexplained variance (total) =	6.00	68.8	68.9
Raw variance unexplained in 1st contrast =	2.33	26.8	39.0
Perseverance			
Total raw variance =	4.82	100.00	100.00
Raw variance explained by measures =	1.82	37.8	37.6
Raw variance explained by persons =	0.60	12.6	12.5
Raw variance explained by items =	1.21	25.2	25.1
Raw unexplained variance (total) =	3.00	62.2	62.4
Raw variance unexplained in 1st contrast =	1.6	35.2	56.6
Decision making			
Total raw variance =	7.73	100.00	100.00
Raw variance explained by measures =	2.73	35.4	35.5
Raw variance explained by persons =	0.97	12.6	12.6
Raw variance explained by items =	1.76	22.8	22.9
Raw unexplained variance (total) =	5.00	64.6	64.5
Raw variance unexplained in 1st contrast =	1.5	19.4	30.0
Learning from mistakes			
Total raw variance =	4.86	100.00	100.00
Raw variance explained by measures =	1.86	38.3	38.6
Raw variance explained by persons =	0.68	14.1	14.3
Raw variance explained by items =	1.17	24.2	17.7
Raw unexplained variance (total) =	3.00	61.7	61.4
Raw variance unexplained in 1st contrast =	1.74	35.9	58.2


*Similar values are expected in the observed and expected raw variance percentages.*

**FIGURE 1:**
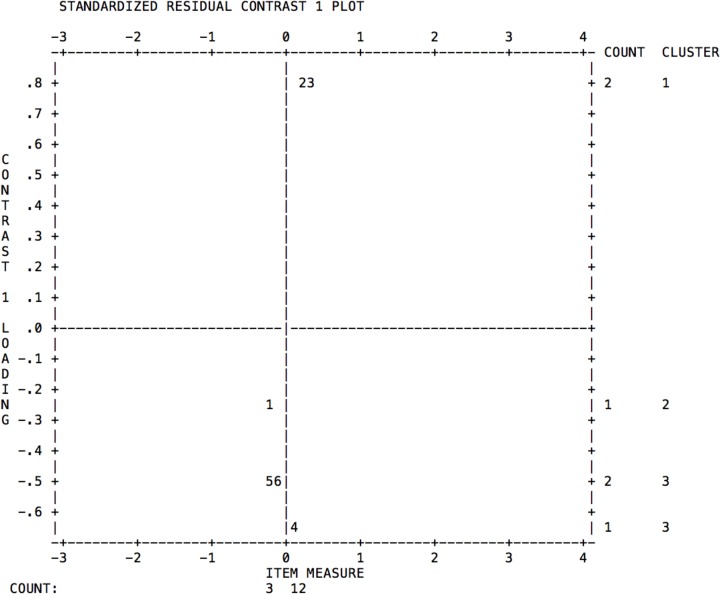
Analysis of the first contrast for the items of the subscale Goal-setting.

The components of the first contrast were analysed for the subscale Goal-setting, with evidence of a possible second dimension. It was found that the behavior of the reverse items is different to the direct items. As can be seen in **Figure 1**, items 2 and 3 (reverse) – corresponding to items 33 and 40, respectively, in the original numeration – appear in a different quadrant and cluster to items 1, 4, 5, and 6 (direct). These reverse items are those that appear to be generating a second dimension underlying the subscale Goal-setting.

In accordance with the procedure followed by Brentari and Golia (2007) in a data simulation study for the detection of unidimensionality using the Rasch model, one should focus on the size of the eigenvalues related to the factors identified through PCAR, and the infit and outfit mean-square values. Therefore, although in the first contrast the Goal-setting scale presents 2.33 eigenvalues, the mean-square fit values of items 33 and 40 (see **Table 6**) are adequate to the model, as are its correlation values. In accordance with Brentari and Golia (2007), the conclusion according to the psychometric evidence would be that these items do not form a separate dimension, since they are connected to the “Goal-setting” dominant latent trait, of which they could be a sub-dimension.

**Table 6:** Infit and outfit estimations for each item by subscale.

			INFIT		OUTFIT		PT-MEASURE	
					
Item	Measure	Model SE	MNSQ	ZSTD	MNSQ	ZSTD	CORR.	EXP.
Goals					
33	0.27	0.04	1.20	4.3	1.18	3.9	0.50	0.55
40	0.24	0.04	1.22	4.7	1.19	4.1	0.51	0.55
42	0.10	0.04	0.84	-3.7	0.83	-3.9	0.58	0.54
1	-0.19	0.04	1.06	1.3	1.12	2.4	0.51	0.52
47	-0.20	0.04	0.79	-4.7	0.78	-4.9	0.59	0.52
49	-0.21	0.04	0.84	-3.6	0.89	-2.3	0.52	0.52
Perseverance					
6	0.33	0.03	1.22	4.7	1.21	4.3	0.54	0.61
41	0.03	0.03	0.98	-0.3	0.96	-1.0	0.61	0.60
34	-0.35	0.04	0.78	-5.3	0.82	-4.0	0.63	0.58
Decision making					
55	0.13	0.04	1.21	4.5	1.21	4.3	0.49	0.59
19	0.07	0.04	0.88	-2.7	0.88	-2.8	0.63	0.59
12	-0.01	0.04	0.94	-1.3	0.95	-1.0	0.58	0.59
5	-0.09	0.04	1.05	1.1	1.02	0.4	0.64	0.59
13	-0.10	0.04	0.93	-1.5	0.94	-1.4	0.61	0.59
Learning from mistakes					
21	0.19	0.04	1.26	5.4	1.23	4.6	0.58	0.64
28	0.15	0.04	1.00	0.0	0.98	-0.4	0.67	0.64
57	-0.34	0.04	0.77	-4.8	0.76	-4.9	0.63	0.60
Mean	0	0.04	1.00	-0.1	0.99	-0.4		
SD	0.24	0	0.18	3.9	0.17	3.5		


*MNSQ (infit and outfit) values between 0.5 and 1.5 in each item are considered to fit the model. The item numeration corresponds to the original test. Column PT-MEASURE CORR. indicates alignment between the item and the ability of the respondent. The correlation values are close to the expectations of the model indicated in column PT-MEASURE EXP. Model SE is the standard error.*

#### Model Fit of the Items by Subscale

Infit and outfit MNSQ values between 0.5 and 1.5 (Bond and Fox, 2012) were taken as indicators of fit values with an expected value of 1. Values higher than 1.5 indicate that the item is erratic, and values below 0.5 indicate that the item is very predictable. Values higher than 2 are a potential threat to the quality of the measure (Linacre, 2002). As the results show (see **Table 6**), all the items of the four subscales present a good fit to the model, since its values are within the parameters established for the MNSQ.

In **Table 7**, it can also be seen that there are no negative correlations between the items and the measure (PT-MEASURE-CORR column) and that the correlation values tend to be moderate and high: the lowest value being 0.49 for item 55, and the highest value 0.67 for item 28. This correlation is an indicator of the correct alignment between the question and the person’s ability: the higher it is the better. Likewise, in the PT-MEASURE-EXP column, it is shown that the correlations observed are very close to model expectations (see **Table 6**).

**Table 7:** Reliability for items and for persons.

	Item reliability	Item separation	Reliability for persons	Separation for persons
Goal-setting	0.97	5.37	0.57	1.14
Perseverance	0.98	7.63	0.30	0.66
Decision-making	0.82	2.12	0.56	1.12
Learning from mistakes	0.98	6.38	0.40	0.81


*Separation index for persons lower than 2, indicates that the instrument is not sentitive enough. Items separation below 3 are considered low.*

#### Reliability of Measure and of Persons

**Table 7** shows the reliability values for persons and items for each of the subscales analysed. In the four subscales, the values are more than adequate for items and low/moderate for persons. The reliability of the items is interpreted as Cronbach’s alpha. Regarding the separation of the items, values lower than 3 are considered low (unlike the results presented in the four subscales). This indicates that the sample is large enough to confirm the hierarchy of difficulty of the items, that is, the construct validity of the instrument (Smith, 2012).

**Table 7** also shows the data of the measure of separation for persons by subscale. An index is considered low in separation for persons with values lower than 2, as with the results presented by the four subscales. This indicates that the instrument is not sensitive enough to identify persons with high and low ability in the variable measured (Smith, 2012).

#### Estimation and Interpretation of the *b* Parameter

The Rasch model establishes the construct validity in accordance with the item hieracrchy, which can be observed in the Wright Map. This map is obtained using item difficulty, and shows the distribution of the items on the right and of persons on the left. The items should form a continuous scale on which low-difficulty items are located lower down, medium-difficulty items in the middle, and high-difficulty items in the upper part. Persons are distributed in the same way, according to their attribute level in the variable measured.

**FIGURE 2:**
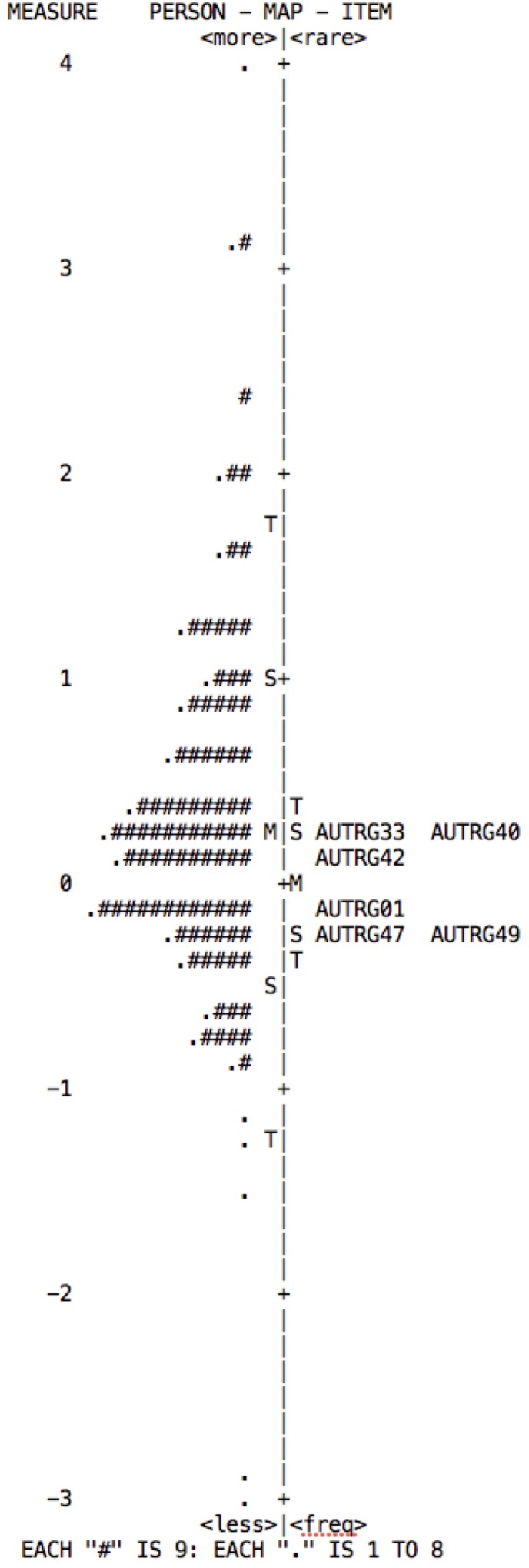
Wright map of persons and items for the Goal-setting subscale. The map indicates that in order to cover the ability range of persons to at least 70%, items of greater difficulty need to be added.

**FIGURE 3:**
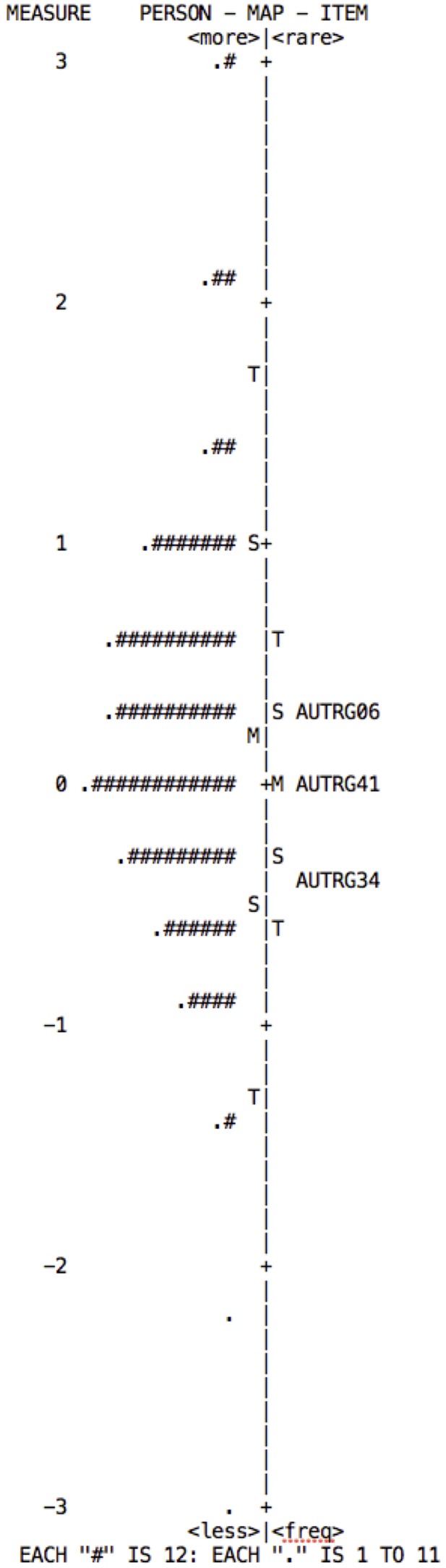
Wright map of persons and items for the Perseverance subscale. The map indicates that in order to cover the ability range of persons to at least 70%, items of greater difficulty need to be added.

**FIGURE 4:**
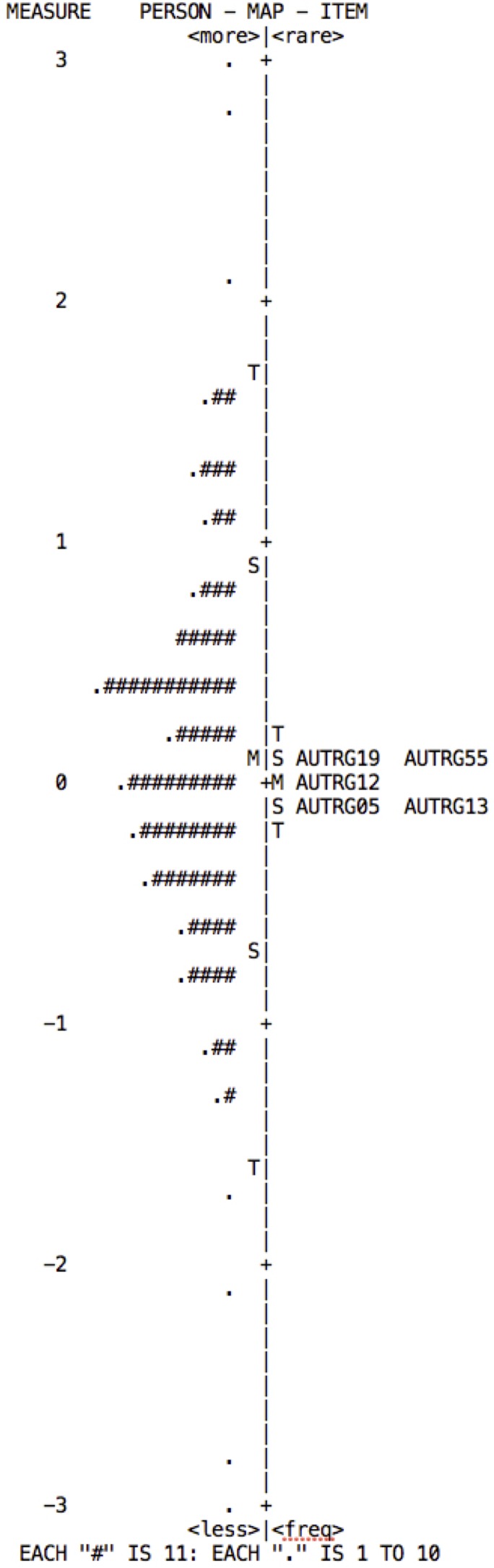
Wright map of persons and items for the Decision-making subscale. The map indicates that in order to cover the ability range of persons to at least 70%, items ofgreater and of less difficulty need to be added.

**FIGURE 5:**
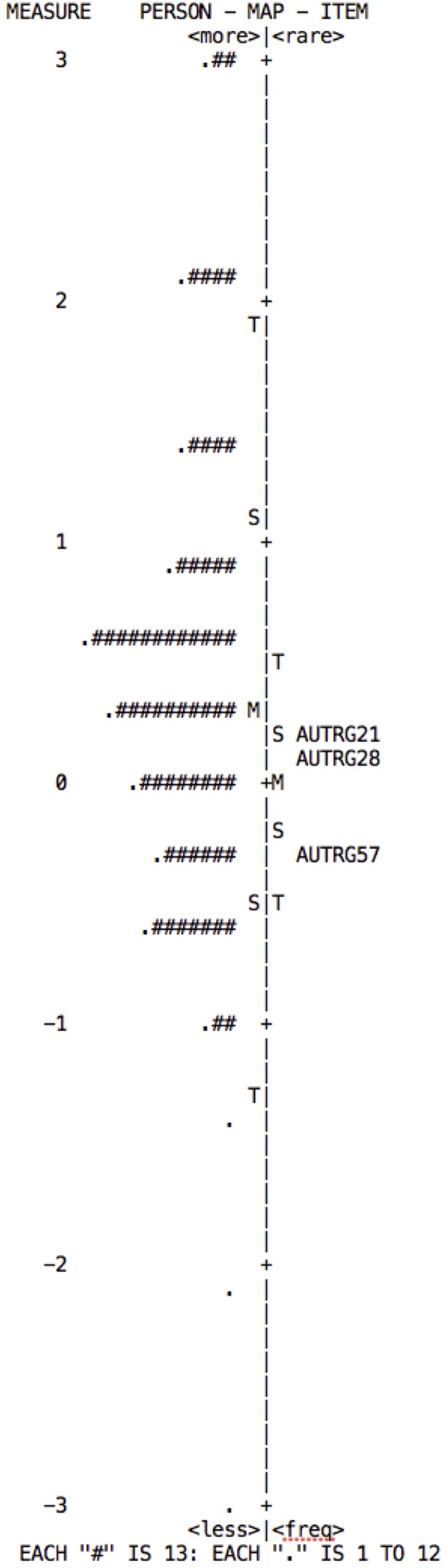
Wright map of persons and items for the Learning from mistakes subscale. The map indicates that in order to cover the ability range of persons to at least 70%, items of medium difficulty and of greater difficulty need to be added.

From the model, one expects: a normal distribution of persons; that there is an alignment between persons and items; and that the items are distributed along the “ruler,” covering at least 70% of the spectrum on which the persons are distributed (Smith, 2012). According to the distribution maps for each of the subscales (**Figures 2–5**), although they show adequate distribution of items these are insufficient to cover the individuals’ range of ability; falling short mainly in the highest levels.

#### Functioning of the Response Categories

**FIGURE 6:**
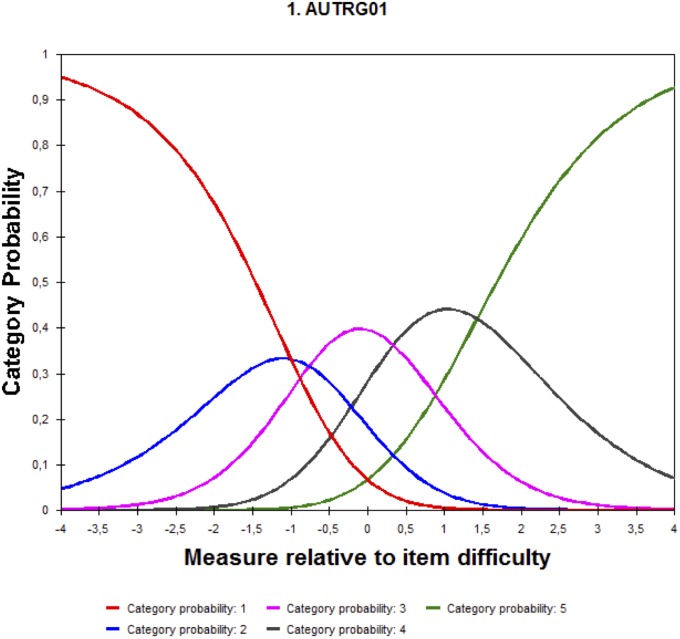
Category probability curves for the subscale Goal-setting.

The response categories for the test are as follows: (1) not at all, (2) somewhat, (3) moderately, (4) quite a lot, and (5) a lot. Using the Rating Scale Model (RSM) for polytomous items, the order of the categories and the clear differentiation between them was verified. The *Category Probability Curves* show that the four subscales present a correct order and differentiation of each category along the attribute measurements (1 to 5). Although the Goal-setting subscale fulfils the requirements of the functioning of the scale, it has little modal differentiation in the response 2 category (see **Figure 6**). The rest of the response categories appear to be clearly differentiated.

#### Analysis of Differential Item Functioning (DIF)

There is empirical evidence of possible DIF when a group of persons do not have the same probability of responding to an item correctly despite having the same attribute level. Measurement invariance was tested for each subscale between men and women (see **Table 8**), and between the four academic years (see **Table 8**).

**Table 8:** Summary of the analysis of the differential item functioning by school year.

Item	Subscale	School years	DIF contrast	*t*	*p*-Value
33	Goals	3–4	0.52	4.33	0.000
21	Learning from mistakes	3–4	0.59	4.20	0.000
28	Learning from mistakes	4–3	0.44	-3.76	0.000
55	Decision-making	3–1	0.36	-3.73	0.000
19	Decision-making	4–3	0.38	-3.21	0.001


*Difficulty should be interpreted for the school year that appears first in the column “School Years.” For example, in the first row, item 33 is easier for Year 3 than for Year 4. DIF contrast is interpreted as an effect size and comes to attention when it is greater than 0.5 and the *t* values are significant.*

The criteria used for establishing possible DIF is that the DIF contrast values are higher than 0.5 logits, which is the difference in the difficulty of an item between the two groups. It was also taken into account whether or not the *t*-values were greater than 2 and if there were significant differences (*p* ≤ 0.05) (Bond and Fox, 2012). Values indicating possible DIF by gender were not found for any subscale. However, some possible DIF values by year are reported in **Table 8**. As can be seen, there are some items that – although they present values lower than a DIF contrast of 0.5 logits – have significant values at *p* ≤ 0.05, greater than 2 in Student’s *t*-test.

## Discussion and Conclusion

### Exploratory and Confirmatory Factorial Analysis

The first objective of this study was to analyze the factorial structure of the SRQ questionnaire (Brown et al., 1999) and its later versions (Carey et al., 2004; Neal and Carey, 2005; Potgieter and Botha, 2009; Pichardo et al., 2014) in use in early and middle adolescence. The CFA results show a better data fit to the model proposed in Pichardo et al. (2014), which has 17 items and 4 factors (goal-setting, perseverance, decision-making, and learning from mistakes). The proportion of variance explained and the internal consistency also showed adequate values. Nonetheless, for the fit of the data to the model, it has been necessary to control the method effect of the reversed items. It appears that the relation between method effect and personality traits are evidence of a response style. Other studies with these characteristics, and that analyze the factorial structure of personal trait measures (DiStefano and Motl, 2006), as well as variables of the “Self,” self-concept and self-esteem, recommend controlling the method effect when the statements are negative or reverse-valued (Tomás et al., 2013).

The method effect of negatively worded items in the CFA and the analysis at item level show that these could be affecting the results and construct validity. Five items out of a total of 17 have low saturation and are not significant with their factor. However, these have been kept in the analysis because the results with a sample of university students were adequate (Pichardo et al., 2014). This effect may be a response style, due to the poorer reading comprehension skills of secondary-school students compared to university students. Therefore, the greater and significant saturation with the method effect factor makes it advisable to revise the way these items are written (items 6, 21, 33, and 40): in particular, to put them in positive terms and make them easier to comprehend for students in early adolescence.

### Rasch Analysis

With Rasch analysis, it is possible to obtain additional psychometric data from the analyzed test: data that it has not been possible to identity with other statistical techniques. Among that additional data, it is worth highlighting the following: (1) to discover if, effectively, the content of the items covers the range of the attribute measured; (2) to identify if the response options from the Likert scale are appropriate; and (3) to discover if the test adequately differentiates people with high/low levels of the attribute. These are several of the advantages reported in the literature on the Rasch model, which allow for tests to be refined, thus improving the evaluation (Heesch et al., 2006), and permitting us to obtain more cost-effective tests each time (Settanni et al., 2015).

With regard to this study’s objective to establish the psychometric properties of the SSRQ for secondary school students using the Rasch model, the results in general indicate a correct fit of the data to the model and evidence the reliability and validity of the measure in its different dimensions, according to the parameters established by the model. However, a detailed examination of the results poses some questions that should be addressed by ensuing studies.

Although the results indicate the existence of a second dimension of the subscale Goal-setting, given that multidimensionality always exists in one way or another, there is a question as to whether the data multidimensionality is so large or so emphatic as to merit dividing the items into two separate tests, in accordance with the first contrast of residuals higher than two eigenvalues. Taking the procedure of Brentari and Golia (2007) into account, there would not be enough evidence to speak of a separate latent trait in the Goal-setting dimension, as opposed to a sub-dimension within the same scale. However, it still needs to be explored whether this apparent multidimensionality is due to a method error originating from the reverse items. Therefore, before thinking about dividing the Goal-setting scale into two dimensions, a procedure similar to that used by Hooper et al. (2013) should be conducted and tested in a similar sample with the reverse items written in a positive sense, in order to establish afresh the psychometric behavior of the items and dimensions.

However, given that the SSSRQ reverse items were shown to behave similarly in a study with a sample of university students (Garzón Umerenkova et al., 2017) – with a possible method effect, but one not as notable as that presented with secondary school students – it does seem that educational level can affect comprehension of and responses to these reverse items. This shows that, as well as revising the reverse questions in accordance with the construct validity evidence presented, the subscales would benefit from the inclusion of items of greater and/or lesser difficulty, to give broader coverage of the ability range shown by the participants.

Consequently, increasing the items would improve the measure in two ways. First, broadening the range of the attribute measured by the whole test and each subscale would improve construct validity. Moreover, reliability for person – which is insufficient for all subscales – would also be improved. Note that, although the reliability for the measure (interpreted as Cronbach’s alpha) is more than adequate, for persons it is not.

Regarding the reliability for persons, it should be pointed out that Rasch analysis provides data for this type of reliability: data that cannot be estimated so clearly using other analysis models. The reliability for persons is important in the context of educational assessment on a practical level, since it gives information on the instrument’s degree of sensitivity or discrimination capacity in differentiating persons according to the degree in which they possess the attribute measured. A minimum separation of 2 is expected, since this means that the instrument can clearly differentiate at least two groups of individuals according to the degree to which they possess the attribute.

Furthermore, when analyzing the possible DIF, 5 of the 17 items (items 33, 21, 28, 55, and 19) present some evidence on a possible differential functioning by school year: four of them between Years 3 and 4, and four of them reverse-scored. But, as the existence of some DIF values does not necessarily imply bias in an item (Wright and Stone, 1999), all the items indicated should be studied further to establish whether there really is bias by school year; and their content should be analysed to establish the possible reason why this evidence is most notable between Years 3 and 4.

The functioning of the response categories appears to be adequate for all subscales. Although the Goal-setting subscale category two (2) is not as differentiated as the other response categories, there is not enough evidence to consider combining it with category three (3).

In summary, the examination of the internal structure (with CFA) and of the psychometric characteristics (with Rasch analysis) concludes that the SSSRQ can be an adequate instrument to evaluate self-regulation in adolescents. This instrument has been used a great deal with university students and can be particularly useful in the study of adolescents’ habits and abuse of alcohol and substances, as well as gambling additctions. In this manner, this instrument could also be converted into a useful tool for the diagnosis, within an educational context, of possible deficits in adolescents’ self-regulation; or to monitor the progress of this variable, after carrying out an intervention aimed at improving it.

The ability to self-regulate has been identified as a key developmental factor that plays a critical role in engaging in risk behaviour, and therefore, it is a target for preventive interventions (Crockett et al., 2006). Detecting those adolescents with low ability to self-regulate makes it possible, on the part of teaching staff and families, to take actions directed at improving their self-regulation and reducing possible problems in adulthood. In this line, Stormshak et al. (2017) conducted a longitudinal study to discover the effects of a program aimed at improving the self-regulation of adolescents within the family context. The results show fewer reports of high-risk behavior during emerging adulthood (socio-emotional risk; sexual behaviour risk; and alcohol, marijuana, and illicit drug use risk) among the subjects partipating in the program. On the other hand, different interventions directed at improving self-regulation within an educational context, with the participation of teaching staff, have been shown to be effective in raising academic performance, problem-solving ability, and motivation (Cleary and Zimmerman, 2004) or in improving quality of life and decreasing behavior problems in the classroom (Matos et al., 2012).

Nevertheless, this study has some limitations, that should be considered for future research. On the one hand, there is no variability in the sample, in which all participants come from the same region. On the other hand, it seems that the instrument was not completely correctly translated and adaptated for early and middle adolescence in a Spanish context. Equally, the results should be contrasted with larger samples, and with samples containing participants from different geographic areas of Spain and other Spanish-speaking countries. Additionally, the sample is comprised only of students in secondary school. Since the SSSRQ is designed to measure general self-regulation in different contexts, including a more diverse sample of adolescents would make it possible to test the characteristics of SSSRQ as an instrument for assessing self-regulation in other contexts (not only academic). For future investigations, it would be interesting to include samples of adolescents who are not attending school – or who are even at risk of social exclusion – in order to test the validity of this instrument with these more diverse sample types.

Taking note of the conclusions and limitations of the study, it is possible to outline some suggestions for advancing the psychometric characteristics of the SSRQ in adolescents, both as an instrument of analysis and as a diagnostic tool of self-regulation in general – and of the dimensions of goal-setting, perseverance, decision-making, and learning from mistakes in particular. For future studies, we would advise rewording the item (and particularly the reversed items) to make them positively worded and easier to understand for participants in early- and mid-adolescence. With the aim of improving the diagnosis of self-regulation, Rasch analysis shows that the items should be broadened, with different degrees of difficulty in the four dimensions.

## Author Contributions

MP contributed in the final writing, review research, and data analysis. FC contributed in the conception and design of the work, review research, and data analysis. AG-U contributed in the data analysis, final writing, and review research. JdA contributed in the coordination of R&D Project, data collection, final writing, and data analysis. FP-S contributed in the review research and data collection. JA-R contributed in the data collection and review research.

## Conflict of Interest Statement

The authors declare that the research was conducted in the absence of any commercial or financial relationships that could be construed as a potential conflict of interest.
